# Automated Coronary Artery Tracking with a Voronoi-Based 3D Centerline Extraction Algorithm

**DOI:** 10.3390/jimaging9120268

**Published:** 2023-12-01

**Authors:** Rodrigo Dalvit Carvalho da Silva, Ramin Soltanzadeh, Chase R. Figley

**Affiliations:** 1 Department of Radiology, Rady Faculty of Health Sciences, University of Manitoba, Winnipeg, MB R3T 2N2, Canada; soltanzr@myumanitoba.ca; 2 Division of Diagnostic Imaging, Health Sciences Centre, Shared Health Manitoba, Winnipeg, MB R3T 2N2, Canada; 3 Biomedical Engineering Program, Price Faculty of Engineering, University of Manitoba, Winnipeg, MB R3T 5V6, Canada

**Keywords:** Voronoi diagram, centerline, coronary artery, Rotterdam coronary artery algorithm evaluation framework, MICCAI coronary artery tracking challenge

## Abstract

Coronary artery disease is one of the leading causes of death worldwide, and medical imaging methods such as coronary artery computed tomography are vitally important in its detection. More recently, various computational approaches have been proposed to automatically extract important artery coronary features (e.g., vessel centerlines, cross-sectional areas along vessel branches, etc.) that may ultimately be able to assist with more accurate and timely diagnoses. The current study therefore validated and benchmarked a recently developed automated 3D centerline extraction method for coronary artery centerline tracking using synthetically segmented coronary artery models based on the widely used Rotterdam Coronary Artery Algorithm Evaluation Framework (RCAAEF) training dataset. Based on standard accuracy metrics and the ground truth centerlines of all 32 coronary vessel branches in the RCAAEF training dataset, this 3D divide and conquer Voronoi diagram method performed exceptionally well, achieving an average overlap accuracy (OV) of 99.97%, overlap until first error (OF) of 100%, overlap of the clinically relevant portion of the vessel (OT) of 99.98%, and an average error distance inside the vessels (AI) of only 0.13 mm. Accuracy was also found to be exceptionally for all four coronary artery sub-types, with average OV values of 99.99% for right coronary arteries, 100% for left anterior descending arteries, 99.96% for left circumflex arteries, and 100% for large side-branch vessels. These results validate that the proposed method can be employed to quickly, accurately, and automatically extract 3D centerlines from segmented coronary arteries, and indicate that it is likely worthy of further exploration given the importance of this topic.

## 1. Introduction

Coronary artery disease (CAD) is defined by the narrowing or blockage of coronary arteries due to the accumulation of cholesterol and fatty deposits along the inner lining of the arterial wall [1] that can reduce blood supply to the heart muscle [2]. CAD is estimated to be the leading cause of mortality in the United States (approximately 61,000 deaths annually) and the third leading cause of mortality worldwide (approximately 17.8 million deaths annually) [3]. The severity and anatomical extent of CAD can be assessed non-invasively using coronary computed tomography angiography (CTA) [1]. Using coronary CTA to extract the coronary artery centerline and lumen is a fundamental step in assessing plaque extent and stenosis area, leading to high-quality models for cardiovascular hemodynamic analysis, functional evaluation, surgical planning, and simulation [4]. However, manual extraction of the centerline and lumen from volumetric coronary CTA images is a time-consuming task and requires expert skills, which has led to the pursuit of computer-aided solutions using automated segmentation and centerline algorithms [5]. In an effort to stimulate the study on coronary artery centerline segmentation, the workshop ’3D Segmentation in the Clinic: A Grand Challenge II—Coronary Artery Tracking’ was introduced at the 11th International Conference on Medical Image Computing and Computer-Assisted Intervention (MICCAI-2008), where authors were given 8 training datasets (with manually labeled reference centerlines) and then invited to submit their results on the 24 test datasets. These datasets were later formally introduced along with a standardized coronary artery centerline evaluation methodology known as the Rotterdam Coronary Artery Algorithm Evaluation Framework (RCAAEF) [6].

Several advanced visualization techniques relying on coronary artery centerlines have been shown to facilitate clinical workflows [7,8,9,10,11]. The categories of coronary artery centerline extraction algorithms can be divided into: (1) automatic extraction methods e.g., [12,13,14,15], which find the centerlines without any user-interaction; (2) extraction with minimal user-interaction e.g., [16,17,18], where only one point per vessel is allowed to be used as an input for the algorithm; and (3) interactive extraction e.g., [19,20,21], which includes any other method that requires more than one point per vessel as input [6].

Among the original 13 algorithms submitted within the grand challenge, the top performer implemented a multiple hypothesis tracking (MHT) framework [19,22], and according to the standardized RCAAEF evaluation criteria, had a mean overlap (OV), overlap until first error (OF), overlap with the clinically relevant part of the vessel (OT), and average inside (AI) of 98.5%, 83.1%, 98.7%, and 0.23 mm, respectively [6]. However, despite performing very well, the MHT method was a category 3 (interactive extraction) algorithm, whereas the top-performing category 1 (fully automatic extraction) algorithm [23] had a substantially lower OV (84.7%), OF (65.3%), and OT (87.0%), and a higher AI (0.28 mm). Therefore, given the obvious advantages of fully automatic coronary artery tracking methods (particularly for potential clinical applications), this has continued to be an active area of exploration, with several more recent approaches reporting promising results that could be used to guide computer-aided diagnostic tools to screen for CAD.

Although several of the more recent automated coronary artery tracking methods are based on deep learning techniques, other approaches have recently been introduced. For example, Liu et al. [24] introduced a new fully automatic model-mapped and inertia-guided minimum path tracking algorithm combining direction features from both atlases and inertia features from previously extracted centerline points. The robustness of this method was evaluated using RCAAEF criteria and was found to have the following characteristics: OV (83.7%), OF (65.6%), OT (85.4%), and AI (0.38 mm). Other methods, such as a Bayesian tracking algorithm based on adaptive particle filters [25], have reported even higher OV (86.2%), OT (92.5%), and lower AI (0.25 mm). However, due to computational complexity, the particle filtering approach required fairly long processing times (approximately 12 min per image) to achieve the best results, which still leave room for accuracy improvements.

With this in mind, the goal of the current study was to test a recently reported automatic 3D Voronoi-based centerline extraction algorithm [26], originally developed as a generic method to skeletonize various complex 3D objects, and use the RCAAEF to validate and benchmark its performance when applied to automated coronary artery tracking. First, the divide and conquer 3D centerline extraction method using Voronoi diagrams is briefly reviewed. Then, a method is described and implemented to generate synthetically segmented coronary artery branches for all 32 vessels included in the RCAAEF training dataset. The segmented coronary artery dataset is then presented to the 3D Voronoi algorithm to extract the centerlines from all 32 vessel branches. The extracted centerlines are then compared to the annotated ground truth RCAAEF centerlines using standardized evaluation metrics (i.e., OV, OF, OT, and AI), and the results are compared to other recently reported fully automated coronary artery centerline extraction methods.

## 2. Materials and Methods

### 2.1. The 3D Centerline Extraction Methodology

**Figure 1:**
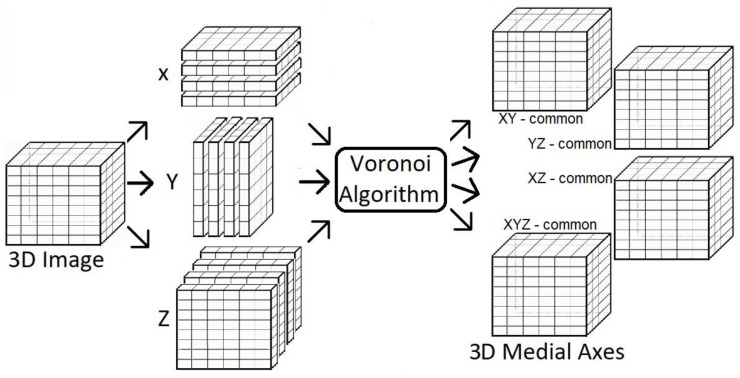
Illustration of the 3D Voronoi-based automatic centerline extraction algorithm pipeline.

In this study, an automatic 3D divide and conquer Voronoi diagram method [26] is applied for coronary artery centerline extraction. The divide and conquer aspect refers to the fact that any 3D image can be partitioned into several 2D images, allowing a 2D Voronoi diagram algorithm to be applied to each 2D image individually. In this approach, a 3D segmentation is first divided into multiple 2D images in the *x*, *y*, and *z* directions. Then, a Voronoi algorithm is applied to each of the generated 2D images to extract centerline points from every slice along each of the planes. The extracted 2D centerlines are then stacked along their corresponding slicing directions to create 3D medial surfaces (i.e., in the X-direction, Y-direction, and Z-direction), and the common medial surfaces are then determined from the intersections between any two (XY-common, YZ-common, XZ-common) directions, as well as the intersections between all three (XYZ-common) directions (Figure 1). Complete theoretical and methodological details of the automated 3D Voronoi-based centerline extraction method itself have been previously described in detail [26], so the remainder of the current manuscript will focus on evaluating its performance when applied for automated coronary artery centerline tracking.

### 2.2. Synthetic Dataset Generation

Data for the current study were acquired from the RCAAEF dataset (http://coronary.bigr.nl/centerlines/ (accessed 19 March 2020)) [6]. The RCAAEF provides 8 publicly available cardiac CTA training images, which each include 4 coronary vessels (i.e., 32 training vessels) with a mean voxel size of approximately 0.32 × 0.32 × 0.4 mm^3^. For each of the training datasets, the four vessels are always defined as right coronary artery (RCA), left anterior descending artery (LAD), left circumflex artery (LCX), and large side-branch of these main coronary arteries. The training data also include files with criterion standard *x*, *y*, and *z* centerline coordinates and radii at each point along each of the 32 annotated vessel branches.

**Figure 2:**
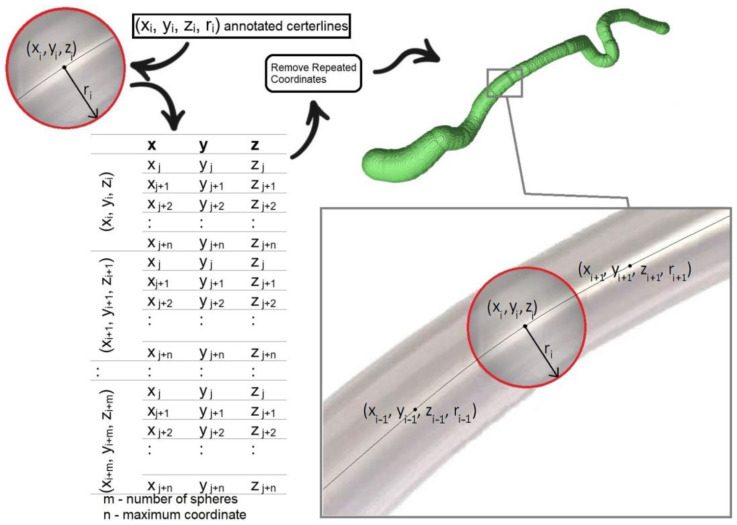
Process to create the synthetic dataset. First, individual spheres are created based on *x*, *y*, *z* coordinates and radii of the RCAAEF annotated centerlines. Points within the union of spheres along the centerline are then converted into one matrix containing *x*, *y*, and *z*. Any repeated coordinates are removed. Remaining coordinates are then used to create a 3D synthetically segmented vessel image.

Since the aforementioned 3D Voronoi-based method can be used to extract centerlines from any complex 3D object, and therefore used in combination with any segmentation approach, the objective of the current study was to focus solely on quantifying coronary artery centerline extraction accuracy (i.e., without potentially introducing segmentation error). Therefore, a synthetic vessel dataset was created using the 32 coronary artery branches included in the RCAAEF training dataset and a previously reported union of spheres approach that seeds a series of spheres with radius *r_p_* at each *x_p_*, *y_p_*, and *z_p_*, at every point (p) along the ground truth centerline [27]. Briefly, the *x*, *y*, and *z* coordinates and radii along the annotated (ground truth) centerline are each rounded to one decimal point to reduce image size and avoid computational overloading. Then, individual spheres (with 200 × 200 faces and radius *r*) are created at each centerline point, and the *x*, *y*, and *z* coordinates of all points within each of the spheres are extracted and saved into a 3-column matrix. After combining coordinates from all of the spheres along the vessel segment, repeated (overlapping) coordinates are removed, and the remaining coordinates are assigned a value of 1 before saving the 3D matrix as a Neuroimaging Informatics Technology Initiative (NIfTI; .nii) image file. This procedure was repeated for all 32 vessel branches included in the publicly available RCAAEF training dataset. Figure 2 illustrates the union of spheres process that was used to produce the synthetically segmented 3D binary vessel segments, and the MATLAB script to perform this operation (using the RCAAEF training data text files) is provided in Appendix A.

### 2.3. Model Performance Evaluation

The RCAAEF [6] outlined four standardized measurements to evaluate coronary artery centerline extraction accuracy compared to ground truth (annotated reference) centerlines, including total overlap (OV), overlap until the first error (OF), overlap with the clinically relevant part of the vessel (OT), and average inside (AI).

OV represents the ability to track the vessel, and only the vessel, and ranges from 0% (i.e., no overlap; having zero extracted centerline points located within one vessel radius of all reference centerline points) to 100% (i.e., perfect overlap; where all extracted centerline points are located within one vessel radius of all reference centerline points, and having no extraneous points). As proposed in the RCAAEF, the OV is defined as:

(1) $$OV=\frac{\left\|{TPM}_{OV}\right\|+\left\|{TPR}_{OV}\right\|}{\left\|{TPM}_{OV}\right\|+\left\|{TPR}_{OV}\right\|+\left\|{FN}_{OV}\right\|+\left\|{FP}_{OV}\right\|}$$

where $\left\|x\right\|$ is the cardinality of a set of points, TPM_OV_ are the true positive points of the centerline being evaluated, TPR_OV_ are the true positive points of the reference standard, FN_OV_ are false negatives, and FP_OV_ are false positives [6].

OF reflects the percentage of the coronary centerline that has been extracted before the first error (i.e., the most proximal point along the reference centerline without an extracted centerline point falling within one vessel radius). The RCAAEF defines the OF as:

(2) $$OF=\frac{\left\|{TPR}_{OF}\right\|}{\left\|{TPR}_{OF}\right\|+\left\|{FN}_{OF}\right\|}$$

where ${TPR}_{OF}$ are the true positive points until the first error and ${(TPR}_{OF}+{FN}_{OF})$ is the total number of points in the reference centerline [6].

OT indicates how well the method can track the portion of the vessel that is assumed to be clinically relevant (i.e., until the most distal portion of the reference vessel having a diameter > 1.5 mm). The RCAAEF defines the OT as:

(3) $$OT=\frac{\left\|{TPM}_{OT}\right\|+\left\|{TPR}_{OT}\right\|}{\left\|{TPM}_{OT}\right\|+\left\|{TPR}_{OT}\right\|+\left\|{FN}_{OT}\right\|+\left\|{FP}_{OT}\right\|}$$

where TPM_OT_ are the true positive points of the centerline, TPR_OT_ are the true positive points of the reference standard, FN_OT_ are false negatives, and FP_OT_ are false positives, among clinically relevant portions of each vessel branch [6].

AI is the average spatial difference (Euclidean distance) between the extracted and reference centerlines for successfully classified, overlapping points (i.e., the mean accuracy of the centerline among correctly extracted points within the vessel).

To evaluate the accuracy of all centerlines in the current study, the Voronoi-generated and RCAAEF reference centerlines were both interpolated and resampled into a set of 1000 uniformly distributed points, and all four RCAAEF metrics were then calculated. The estimated centerline points from the 3D Voronoi-based algorithm, which fell within a preset distance (i.e., less than or equal to the vessel radius) from the corresponding RCAAEF reference centerline, were labeled as true-positive points of the method (TPM). The remaining points located further than the corresponding reference centerline vessel radius were regarded as false-positive points (FP). Further, the RCAAEF considers the sample points in the reference that are within a preset distance (i.e., less than or equal to the vessel radius) from the target centerline to be true positive points of the reference (TPR). The remaining points of the reference are regarded as false negative (FN).

All of the computational analyses, including the creation of the 3D synthetic dataset, the Voronoi-based 3D centerline extraction, and the calculation of performance evaluation metrics were performed in MATLAB R2021b software on an Intel Core i5-1235U (i.e., 2 performance cores @ 1.3 GHz base, up to 4.4 GHz turbo frequency; 8 efficient cores @ 0.9 GHz base, up to 3.3 GHz turbo frequency) laptop with 16 GB of RAM.

## 3. Results

The synthetically segmented coronary artery datasets (NIfTI files) were presented to the 3D method described in Section 2.1 and XY, YZ, XZ, and XYZ-common centerlines were extracted for each vessel segment. Table 1 shows the size of each of the synthetic images and the 3D Voronoi algorithm centerline extraction time. XZ was the fastest approach with an average centerline extraction time of 47.26 s, followed by YZ (49.08 s), XY (69.16 s), and XYZ (82.73 s).

**Figure 3:**
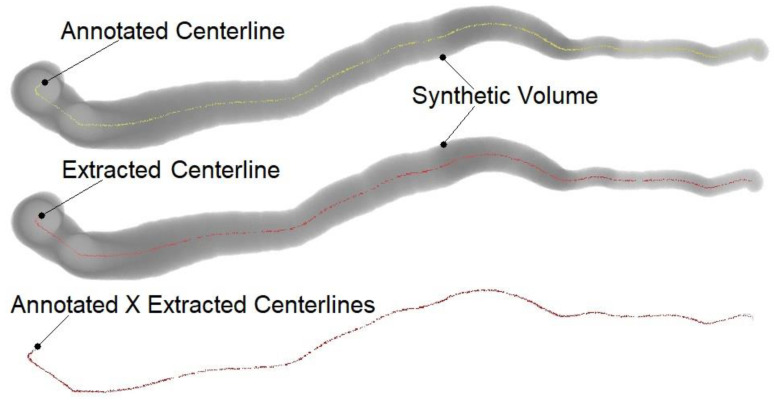
Example of ground truth (RCAAEF annotated) and XY-common extracted centerlines from the RCA branch (vessel 1) in dataset 0.

Table 2 presents the algorithm’s performance results (i.e., OV, OF, OT, and AI) for each of the automatically extracted XY, YZ, XZ, and XYZ centerlines; and Figure 3 depicts an example of the RCAAEF ground truth (annotated) and algorithmically extracted centerlines within the first synthetically segmented coronary artery branch (i.e., vessel 1 of dataset 0). From Table 2, it can be seen that XY yielded the highest OV (99.98%), XY, YZ, and XYZ produced a perfect OF (100%), XY had the best OT (99.99%), and XYZ the shortest distance AI (0.13 mm). For the synthetic dataset, one pixel is represented by a mean of approximately 0.10 mm indicating that all four extracted centerlines (XY, YZ, XZ, and XYZ) yielded AI values that were less than two voxels from the reference centerlines.

Moreover, Table 3 displays the average performance (across all eight datasets) of the algorithmically extracted XY, YZ, XZ, and XYZ centerlines, broken down by vessel type: namely, the RCA (vessel 0), LAD (vessel 1), LCX (vessel 2), and large-size branch (vessel 3). Although all of the centerlines yielded excellent performance (i.e., at or approaching 100%), the XY centerline produced the highest average OV values for the RCA (99.9%), LAD (100%), and LCX (99.96%), while the YZ centerline produced the highest average value for the large-branch vessel segmentations (100%).

## 4. Discussion

To place the current findings in context, we sought to compare them to other recently reported fully automatic coronary artery centerline extraction methods that were (1) benchmarked using the RCAAEF training dataset; and (2) found to have better performance than the best fully automatic method in the original challenge [6] on at least one standard accuracy measure (i.e., OV > 84.7%, OF > 65.3%, OT > 87.0%, and/or AI < 0.28 mm). Upon literature reviews in Pubmed and Google Scholar, eight other peer-reviewed papers were identified that fit these criteria, dating back to the beginning of 2016 (i.e., published in the past 7.5 years). The performance of these methods is summarized in Table 4.

Among these, Han et al. [28] used stochastic geometric processes with active branch searches to simultaneously reconstruct centerlines from the whole coronary artery tree. Based on this, they found 81.4% (OV), 77.3% (OF), and 87.8% (OT) in the RCAAEF training dataset, and a slightly higher OV (84.3%) in the testing dataset. Around the same time, Cui et al. [27] implemented a hybrid approach combining a fast marching and a Runge–Kutta-based method, which was validated using RCAAEF training data from a single CTA image (i.e., four vessel branches). Based on the same synthetic vessel segmentation employed in the current study, their method yielded average accuracies of 98.00% (OV), 95.00% (OF), and 97.55% (OT) across the four vessels. Another framework for extracting coronary centerlines by the same author used gradient vector flow (GVF) fields and fast marching to implement a wavefront propagation technique for centerline branch tracking [29]. In that study, 17 segments of the RCAAEF were reconstructed and used to validate the centerline extraction algorithm. The averages of the GVF method were 98.2% (OV), 91.7% (OF), and 98.3% (OT). Alternatively, Salehi et al. [30] combined a continuous complex wavelet transformation (CCWT) to minimize the influence of noise, a compound pre-processing approach to improve image quality and remove unnecessary image regions, an enhanced tubular tracking strategy based on 3D template matching, and a newly proposed branch searching algorithm that seeks additional branches through local region growing and morphological skeletonization. They did not report OV and OF separately for the training and testing RCAAEF datasets, but their average across the eight training datasets was 99.01% (OT) and 0.27 mm (AI), and the pooled averages across all 32 training and testing datasets were reported as 85.2% (OV), 75.7% (OF), and 98.5% (OT). Notably, all three of the latter studies reported OT values (i.e., overlap with the clinically relevant portions of the vessels, with a diameter > 1.50 mm) higher than 97.5%.

Although it did not perform quite as well as some of the others, another method [4] introduced a multi-model directional fast-marching strategy that gave training data accuracies of 83.5% (OV), 57.8% (OF), 87.1% (OT), and 0.48 mm (AI); and, like the earlier paper by Han et al. [28], they also reported a higher OV (86.6%) for the RCAAEF testing dataset.

With advances in computational power, novel deep learning approaches have also been presented. For instance, Wolterink et al. [5] recently introduced an automated coronary artery tracking method using a convolutional neural network (CNN) orientation classifier, which was both very quick (approximately 20 s per CTA image) and highly accurate, producing 95.7% (OV), 87.1% (OF), 97.1% (OT), and 0.23 mm (AI). Based on a 3D dilated CNN and double deep q-network (DDQN), Zhang et al. [31] have developed a branch-aware coronary centerline extraction (BACCE) method consisting of two parts: a DDQN-based tracker that accurately predicts the next action of an agent tracing the centerline, and a branch-aware detector that detects the branch points and radius of the coronary artery, allowing the BACCE to automatically trace the branches. Their experimental findings showed that the approach achieved 96.2% (OV), 88.3% (OF), 96.5% (OT), and 0.21 mm (AI) across the eight RCAAEF training datasets. An even more recently introduced tracking method by Jeon [32], combined a deep CNN and particle filter to identify the trajectories from the coronary ostium to each distal end from 3D CTA images. This method obtained an average of 92% (OV), 93% (OT), and 0.36 mm (AI).

Table 4 also presents a comparison between the currently evaluated 3D Voronoi method XY, YZ, XZ, and XYZ centerline accuracies and the previously reported methods that were also benchmarked using the RCAAEF training dataset. Based on this, the automatic Voronoi-based method appears to have the highest reported OV values (especially XY with 99.98%), OF values (especially XY, YZ, and XYZ with 100%), and OT values (especially XY with 99.99%), as well as the lowest AI values (especially XYZ with 0.13 mm). As indicated in the table, some of these studies were based on real-world segmentation of the RCAAEF training data (meaning that the results could include both segmentation and centerline extraction errors), while others used synthetically segmented data (similar to the current study). Nonetheless, this comparison suggests that the 3D divide and conquer Voronoi method is a highly robust approach for coronary artery centerline mapping.

**Table 4:** Comparison of the Voronoi-based method with other recently published algorithms that were benchmarked using the Rotterdam coronary CTA training dataset. The highest reported OV, OF, and OT values (and lowest reported AI values) are indicated in bold font.

Centerline	Segmentation Method	Training Dataset Accuracy				Testing Dataset Accuracy			
Method	(Number of Training Set Vessels)	OV	OF	OT	AI	OV	OF	OT	AI
**Voronoi method (XY)**	Synthetic (32 vessels)	**99.98**	**100**	**99.99**	0.17	-	-	-	-
**Voronoi method (YZ)**	Synthetic (32 vessels)	99.76	**100**	99.75	0.18	-	-	-	-
**Voronoi method (XZ)**	Synthetic (32 vessels)	99.79	99.97	99.87	0.19	-	-	-	-
**Voronoi method (XYZ)**	Synthetic (32 vessels)	99.97	**100**	99.98	**0.13**	-	-	-	-
Jeon [32] (Deep-PF)	Real (32 vessels)	92.00	-	93.00	0.36	-	-	-	-
Zhang et al. [31]	Real (32 vessels)	96.20	88.30	96.50	0.21	-	-	-	-
Wolterink et al. [5]	Real (32 vessels)	95.70	87.10	97.10	0.23	93.70	-	-	-
Jia et al. [4] (MM-DFM)	Real (32 vessels)	83.50	57.80	87.10	0.48	86.60	-	-	-
Salehi et al. [30] (CCWT)	Real (32 vessels)	-	-	99.01	0.27	-	-	98.39	0.26
Cui et al. [29] (GVFFM)	Synthetic (17 vessels)	98.20	91.70	98.30	0.25	95.20	-	-	-
Cui et al. [27] (DTFM)	Synthetic (4 vessels)	98.00	95.00	97.55	-	-	-	-	-
Han et al. [28] (AS)	Real (32 vessels)	81.40	77.30	87.80	-	84.30	-	-	-

One interesting difference in the 3D Voronoi algorithm compared to the other approaches is that OF values were uniformly higher (not lower) than the OV and OT metrics (Table 4); and this was not only a general trend, but was also observed at the level of individual vessel segments (Table 1). To calculate OF, the first error is defined as the first false negative point “when traversing from the start of the reference standard to its end while ignoring false negative points in the first 5 mm of the reference standard” [6]. The RCAAEF reasoned that errors within the first 5 mm can be ignored “because of the strictness of this measure” and because “for some applications [they are] not of critical importance” [6]. Therefore, while OF values lower than OV indicate false negative errors occurring in the middle of a vessel’s vector, OF values higher than OV imply very good tracking along the main portion of the vessel, and that errors are either disproportionately located at the very beginning of the vessel (i.e., within the first 5 mm) or the presence of false positive errors beyond the reference standard (which contribute to OV but not OF). For this reason, especially in extreme cases with very high OV values, it is possible to have 100% OF and lower (albeit still very high) OV when false negative errors only occur within the first 5 mm of the vessel—as is the case for the current 3D Voronoi-based method.

Similarly, because AI is only calculated among correctly classified centerline points, it (by definition) has a lower-bound of zero (0 mm), and an upper-bound equal to the average radius of the vessel segment. The rationale for defining AI this way is explained within the RCAAEF [6]; however, this makes it possible for any given centerline method to simultaneously yield poor (low) OV values and good (low) AI values—i.e., high spatial accuracy over a small portion of the vessel segment. Conversely, the simultaneously high OV values (99.76–99.98%) and low AI values (0.13–0.19 mm) observed for the Voronoi-based centerline method indicate uniformly excellent centerline accuracy over practically the entire length of each vessel segment.

### 4.1. Study Limitations

One limitation of the 3D Voronoi-based divide and conquer approach itself is that larger images generally require longer centerline extraction times, with the RCA and LAD coronary branches typically taking the longest (Table 1). This suggests that future studies, especially those with higher resolution CTA images (and higher resolution coronary artery segmentations), would benefit greatly from the combination of: (1) higher-performance computational resources—particularly more CPUs (and potentially GPUs) to support parallel computing, and (2) compiling the MATLAB code into a faster and more efficient programing language such as C++ (in which optimized executables run approximately 10x faster than in MATLAB) [33]. Together, these factors could realistically be anticipated to reduce the average centerline extraction times with this method from approximately 1 min (Table 1) to somewhere on the order of 1 s or less using a high-performance GPU image processing workstation and an optimized C++ code. The primary goal of the current manuscript was to validate and benchmark the accuracy of the 3D Voronoi-based method for automatic coronary artery centerline extraction; however, with that objective achieved, reducing the processing time will certainly be important in its future translation from basic research to clinical applications.

However, perhaps the biggest limitation of the current study was the use of synthetically segmented RCAAEF data. However, after careful consideration, this approach was ultimately chosen because the 3D Voronoi-based centerline algorithm can be used in combination with (i.e., following) any coronary artery segmentation method. Therefore, synthetic segmentation was applied in order to specifically test the 3D centerline extraction accuracy without any potential confounds resulting from segmentation errors. Since the inputs to this centerline algorithm are binary segmentations, it is not inherently sensitive to the CTA image features (e.g., tissue contrast, image artifacts, noise, etc.) per se. However, these and other factors do influence real-world segmentation accuracy, which could then become a limiting factor in the overall centerline extraction pipeline—which we were not evaluating in the current study. Nevertheless, to promote reproducibility (and facilitate direct comparisons in future studies), we have provided the MATLAB code, to recreate all 32 synthetically segmented vessel branches using the publicly available RCAAEF training dataset, in Appendix A. The excellent performance of the 3D Voronoi algorithm, as reported in this study, does suggest that centerline extraction accuracy in real-world coronary artery tracking applications is likely to be very good, and that overall accuracy may indeed be limited by the initial segmentation accuracy. The ideal solution would therefore be to pair the Voronoi-based centerline extraction algorithm with the best available coronary artery segmentation method for optimal overall performance and, in this regard, not having the segmentation and centerline methods inextricably linked is ostensibly an advantage.

### 4.2. Future Directions

Given that the automatic 3D divide and conquer Voronoi-based centerline extraction method [26] exhibited such high accuracy when applied to synthetically segmented coronary artery branches, this opens several potential lines for future research and development.

First, to address some of the limitations described above, the existing in-house MATLAB code should be re-programmed and optimized for parallel (multi-core CPU and GPU) processing in a more efficient computer language (e.g., C++) to facilitate substantial reductions in centerline extraction times using high-performance image processing workstations.

Second, since this centerline approach is independent of the preceding segmentation procedure, testing it in combination with various ‘real-world’ coronary artery segmentation approaches will be important to further evaluate and substantiate its overall clinical utility.

Third, since sufficiently accurate centerlines can be leveraged to quantify other coronary artery features (e.g., overall cross-sectional area, lumen cross-sectional area, etc.) along vascular trajectories [34], this centerline approach could be investigated in combination with 3D vessel and lumen segmentations based on multi-modal imaging data (e.g., combining modern CT angiography, MR angiography, and/or black-blood MRI methods) to evaluate its ability to automatically quantify atherosclerotic features such as coronary luminal narrowing.

Finally, since this automated 3D Voronoi-based centerline algorithm is based on a highly generalizable, bottom-up geometric approach, with nothing inherently specific to coronary arteries, its performance here suggests that it could potentially be used for automated centerline extraction in other medical imaging applications as well (as long as manual or automatic 3D segmentations are available). Therefore, it could be evaluated for potential use in other (non-coronary) vascular imaging applications, and perhaps even automated centerline extractions of non-vascular 3D segmented anatomical structures (e.g., bowel, tumor, brain white matter fascicles, etc.) with complex 3D trajectories.

## 5. Conclusions

To summarize, this study validated and benchmarked a 3D divide and conquer Voronoi-based method to automatically extract 3D centerlines from synthetically segmented versions of the 32 coronary artery branches included in the well-known and widely used RCAAEF training dataset. To the best of our knowledge, this technique demonstrated the highest reported RCAAEF accuracy metrics to date among fully automatic 3D coronary artery tracking methods, yielding an average overlap accuracy (OV) of 99.97%, an overlap until first error (OF) of 100%, an overlap of the clinically relevant portion of the vessel (OT) of 99.98%, and an average error distance inside the vessels (AI) of only 0.13 mm. It therefore appears that this method can be used (in combination with any sufficiently accurate coronary artery segmentation approach) to perform accurate and automatic coronary artery centerline tracking and could perhaps also be used for centerline extraction in other medical imaging applications.

## Figures and Tables

**Table 1:** Rotterdam CTA synthetic image sizes and 3D Voronoi algorithm centerline extraction times.

Dataset	Image	Computational Time (Seconds)			
(Vessel)	Size	XY	YZ	XZ	XYZ
0(0)	1056 × 650 × 609	218.48	148.09	143.75	254.95
0(1)	475 × 685 × 179	15.81	17.32	15.45	24.26
0(2)	842 × 402 × 332	25.96	21.23	25.03	36.10
0(3)	619 × 258 × 139	9.14	8.89	11.00	14.51
1(0)	915 × 830 × 675	212.24	136.12	152.67	250.46
1(1)	441 × 877 × 935	133.83	120.82	53.11	153.87
1(2)	721 × 185 × 577	19.97	12.48	21.43	26.93
1(3)	691 × 489 × 469	30.33	27.64	29.18	43.57
2(0)	531 × 687 × 582	54.95	42.43	41.53	69.44
2(1)	422 × 587 × 843	51.87	46.65	34.79	66.65
2(2)	426 × 494 × 436	25.36	22.50	20.92	34.38
2(3)	529 × 288 × 317	12.35	10.91	13.27	18.26
3(0)	863 × 767 × 678	153.67	98.73	112.10	182.23
3(1)	742 × 901 × 739	190.47	136.74	97.51	212.35
3(2)	807 × 353 × 630	41.98	23.50	36.88	51.17
3(3)	770 × 355 × 279	14.95	12.49	17.57	22.50
4(0)	872 × 606 × 763	135.94	70.00	105.20	155.56
4(1)	588 × 643 × 902	100.41	71.77	56.31	114.24
4(2)	447 × 500 × 753	29.69	23.12	19.84	36.31
4(3)	736 × 265 × 409	13.63	9.53	15.03	19.09
5(0)	438 × 599 × 579	31.26	26.29	22.48	40.01
5(1)	630 × 747 × 861	134.86	102.24	74.33	155.70
5(2)	516 × 324 × 335	12.82	10.72	13.40	18.46
5(3)	662 × 341 × 192	10.25	12.72	16.16	19.56
6(0)	801 × 683 × 718	124.02	76.51	88.85	144.69
6(1)	709 × 809 × 727	134.48	97.65	77.94	155.02
6(2)	509 × 327 × 546	18.05	13.27	15.73	23.52
6(3)	724 × 610 × 381	29.07	25.52	26.85	40.71
7(0)	816 × 369 × 803	75.40	40.13	61.44	88.48
7(1)	639 × 666 × 943	138.07	93.55	78.38	154.98
7(2)	304 × 311 × 330	7.66	5.85	7.00	10.25
7(3)	506 × 126 × 283	6.28	5.01	7.14	9.21
Average	215,321,960 voxels (equivalent to 600 × 599 × 599)	69.16	49.08	47.26	82.73

**Table 2:** Evaluation of the XY, YZ, XZ, and XYZ centerline accuracies from the synthetically segmented Rotterdam Coronary Artery Algorithm Evaluation Framework training dataset (8 CTA images with 4 vessels each) using OV, OF, OT, and AI.

Dataset	XY				YZ				XZ				XYZ			
(Vessel)	OV	OF	OT	AI	OV	OF	OT	AI	OV	OF	OT	AI	OV	OF	OT	AI
0(0)	100	100	100	0.09	99.97	100	100	0.10	99.47	100	100	0.08	99.97	100	100	0.09
0(1)	99.99	100	99.99	0.19	99.99	100	99.99	0.12	99.52	100	99.51	0.31	99.99	100	99.99	0.10
0(2)	100	100	100	0.16	100	100	100	0.17	100	100	100	0.14	100	100	100	0.13
0(3)	100	100	100	0.22	100	100	100	0.09	99.75	100	99.75	0.30	99.95	100	99.95	0.17
1(0)	100	100	100	0.22	99.99	100	100	0.09	98.98	100	99.68	0.15	100	100	100	0.08
1(1)	100	100	100	0.23	99.60	100	99.78	0.13	100	100	100	0.20	99.96	100	99.96	0.17
1(2)	100	100	100	0.07	100	100	100	0.05	100	100	100	0.07	100	100	100	0.06
1(3)	99.70	100	100	0.12	100	100	100	0.05	100	100	100	0.07	100	100	100	0.16
2(0)	99.99	100	100	0.07	99.91	100	100	0.11	100	100	100	0.07	99.99	100	100	0.06
2(1)	100	100	100	0.07	99.04	100	98.24	0.12	98.87	98.9	100	0.10	100	100	100	0.06
2(2)	100	100	100	0.23	99.98	100	99.97	0.60	100	100	100	0.37	99.92	100	99.92	0.19
2(3)	99.99	100	99.99	0.28	100	100	100	0.23	98.91	100	98.91	0.73	99.99	100	99.99	0.34
3(0)	100	100	100	0.08	99.89	100	100	0.14	100	100	100	0.08	100	100	100	0.07
3(1)	99.99	100	100	0.08	100	100	100	0.06	99.99	100	100	0.07	99.99	100	100	0.06
3(2)	99.96	100	100	0.06	100	100	100	0.23	99.97	100	99.97	0.19	99.96	100	99.97	0.17
3(3)	100	100	100	0.20	100	100	100	0.13	100	100	100	0.05	100	100	100	0.046
4(0)	99.99	100	99.99	0.33	95.73	100	95.73	0.35	100	100	100	0.35	99.89	100	99.89	0.28
4(1)	100	100	100	0.21	100	100	100	0.20	100	100	100	0.19	100	100	100	0.18
4(2)	100	100	100	0.16	99.46	100	99.46	0.26	100	100	100	0.15	100	100	100	0.15
4(3)	99.96	100	99.96	0.18	100	100	100	0.13	99.96	100	99.96	0.17	99.96	100	99.96	0.14
5(0)	100	100	100	0.09	100	100	100	0.06	100	100	100	0.06	100	100	100	0.06
5(1)	100	100	100	0.29	100	100	100	0.20	99.99	100	99.99	0.18	99.99	100	99.99	0.18
5(2)	100	100	100	0.06	100	100	100	0.06	100	100	100	0.06	99.87	100	100	0.05
5(3)	100	100	100	0.33	100	100	100	0.14	100	100	100	0.20	100	100	100	0.16
6(0)	99.99	100	100	0.11	100	100	100	0.07	100	100	100	0.08	100	100	100	0.067
6(1)	100	100	100	0.26	99.58	100	99.58	0.39	100	100	100	0.18	100	100	100	0.18
6(2)	100	100	100	0.20	99.29	100	99.29	0.54	97.97	100	97.97	0.66	100	100	100	0.15
6(3)	100	100	100	0.35	100	100	100	0.19	100	100	100	0.19	100	100	100	0.18
7(0)	99.94	100	100	0.08	100	100	100	0.07	99.99	100	100	0.08	100	100	100	0.07
7(1)	99.99	100	99.99	0.26	100	100	100	0.26	99.99	100	99.99	0.27	99.99	100	99.99	0.24
7(2)	99.74	100	99.74	0.13	100	100	100	0.12	99.98	100	99.98	0.15	99.64	100	99.64	0.13
7(3)	99.99	100	99.99	0.16	100	100	100	0.15	99.99	100	99.99	0.14	99.99	100	99.99	0.11
Average	99.98	100	99.99	0.17	99.76	100	99.75	0.18	99.79	99.97	99.87	0.19	99.97	100	99.98	0.13

**Table 3:** Average XY, YZ, XZ, and XYZ centerline accuracies for RCA, LAD, LCX, and large Branch coronary artery segments.

	XY				YZ				XZ				XYZ			
	OV	OF	OT	AI	OV	OF	OT	AI	OV	OF	OT	AI	OV	OF	OT	AI
**RCA**	99.99	100	100	0.13	99.44	100	99.47	0.12	99.81	100	99.96	0.12	99.98	100	99.99	0.10
**LAD**	100	100	100	0.20	99.77	100	99.70	0.18	99.80	99.86	99.94	0.19	99.99	100	99.99	0.15
**LCX**	99.96	100	99.97	0.13	99.84	100	99.84	0.26	99.74	100	99.74	0.22	99.93	100	99.94	0.13
**Large Branch**	99.96	100	99.99	0.23	100	100	100.00	0.14	99.83	100	99.83	0.23	99.99	100	99.99	0.16
Average	99.98	100	99.99	0.17	99.76	100	99.75	0.18	99.79	99.97	99.87	0.19	99.97	100	99.98	0.13

## Data Availability

The synthetically segmented coronary artery datasets presented in this study were generated using the MATLAB code provided in Appendix A, along with the publicly available Rotterdam Coronary Artery Algorithm Evaluation Framework training dataset from the MICCAI 2008 Coronary Artery Tracking Challenge (CAT08; which was previously available online: http://coronary.bigr.nl/centerlines/ (accessed 19 March 2020)). However, since the website is not currently accessible at the time of writing—suggesting that the CAT08 dataset may no longer be publicly available—the 32 synthetic coronary artery segmentations (.nii images) used in the current study can be made available upon reasonable request to the corresponding author(s).

## Appendix A

MATLAB script to generate synthetically segmented vessels from the Rotterdam coronary artery algorithm evaluation framework training data.

clear all, close all, clc

% *.txt file containing x,y,z, and radius (designed for Rotterdam dataset)

[file,pathfile] = uigetfile('*.*');

if isequal(file,0)

   disp('User selected Cancel');

else

   disp(['User selected ', fullfile(pathfile,file)]);

end

fullfilename = fullfile(pathfile, file)

referencein = load(fullfilename);

reference = referencein(:,1:4);

%% DATA ROUNDING

q = 10; reference = round(q*reference); % q = 10 - coarse, 1000 - finest (finest requires more computational power)

%% DATA TRANSLATION

reference(:,1) = reference(:,1) - min(reference(:,1)) + max(reference(:,4)) + 1;

reference(:,2) = reference(:,2) - min(reference(:,2)) + max(reference(:,4)) + 1;

reference(:,3) = reference(:,3) - min(reference(:,3)) + max(reference(:,4)) + 1;

%% SPHERE CREATION

r = reference(:,4); c = reference(:,1:3);

[x y z] = sphere(200,200);

X = reshape((x * r(1,:) + c(1,1)),1,[])';

Y = reshape((y * r(1,:) + c(1,2)),1,[])';

Z = reshape((z * r(1,:) + c(1,3)),1,[])';

parfor ct = 2:length(c) % can be run with a regular for loop, but will take longer

    X = [X; reshape((x * r(ct,:) + c(ct,1)),1,[])'];

    Y = [Y; reshape((y * r(ct,:) + c(ct,2)),1,[])'];

    Z = [Z; reshape((z * r(ct,:) + c(ct,3)),1,[])'];

end

A = unique([X Y Z],'rows'); % remove any overlapping points

A = uint16(A);

clearvars -except A r c

%% IMAGE CREATION

M = uint16([max(A(:,1)), max(A(:,2)), max(A(:,3))]);

l = length(A(:,1));

for k = 1:M(:,3)

    I = zeros(M(:,1),M(:,2));

    for j = 1:l

        if A(j,3) == k

            x = A(j,1); y = A(j,2); I(x,y) = 1;

        end

    end

    Im(:,:,k) = double(imfill(I,'holes'));

end

%% PAD IMAGES WITH 0 VALUE (add zero faces)

Im = padarray(Im,[1 1 1],0,'both');

volshow(Im)               % show volume

%% SAVE NIfTI IMAGE AND WORKSPACE OUTPUTS

Ref = [c r];

[file1,path1] = uiputfile('*.nii','Save Nifti file name');

[file2,path2] = uiputfile('*.mat','Save new coordinate');

[file3,path3] = uiputfile('*.mat','Save .mat file');

filename1 = fullfile(path1,file1);

filename2 = fullfile(path2,file2);

filename3 = fullfile(path3,file3);

niftiwrite(Im,filename1)

save(filename2,'Ref')

save(filename3,'Im','-v7.3') % this .mat file will be very large and requires –v7.3
